# The dynamics of political and affective polarisation: Datasets for Spain, Portugal, Italy, Argentina, and Chile (2019-2022)

**DOI:** 10.1016/j.dib.2023.109219

**Published:** 2023-05-09

**Authors:** Mariano Torcal, Emily Carty, Josep Maria Comellas, Oriol J. Bosch, Zoe Thomson, Danilo Serani

**Affiliations:** a Universitat Pompeu Fabra, Plaça de la Mercè, 08002, Barcelona, Spain; b Universidad de Salamanca, Campus Miguel de Unamuno s/n, 37007 Salamanca, Spain; c Universitat Pompeu Fabra, Plaça de la Mercè, 08002, Barcelona, Spain & Universitat Autònoma de Barcelona, Plaça Cívica, 08193 Bellaterra, Barcelona, Spain; d The London School of Economics, Houghton St, London WC2A 2AE, United Kingdom

**Keywords:** Dataset, Affective polarisation, Political trust, Voting behaviour, Democracy

## Abstract

The TRI-POL project explores the triangle of interactive relationships between affective and ideological polarisation, political distrust, and the politics of party competition. In this project there are two complementary groups of datasets with individual-level survey data and digital trace data collected in five countries: Argentina, Chile, Italy, Portugal and Spain. These datasets are comprised of three waves carried out over a six-month period between late September 2021 and April 2022. In addition, the survey datasets include a series of experiments embedded in the different waves that examine social exposure, polarisation framing, and social sorting. The digital trace datasets include variables on individuals’ behaviours and exposure to information received via digital media and social media. This data was collected using a combination of tracking technologies that the interviewees installed in their different devices. This digital trace data is matched with the individual-level survey data. These datasets are especially useful for researchers who wish to explore dynamics of polarisation, political attitudes, and political communication.

**Specifications Table**

**Table:** 

Subject	Sociology and Political Science
Specific subject area	Public opinion
Type of data	Table, Matrix
How the data were acquired	Three-wave online panel survey and digital trace data from a sample of the voting-age population in Spain, Portugal, Italy, Argentina, and Chile. One group of datasets consist of survey data from these respondents. Original survey experiments were embedded into each of the three waves of the survey. Individuals were randomly selected to form the different treatment groups. Another group of datasets consist of digital trace data on those individuals’ online behaviours on digital and social media websites and applications.
Data format	Raw survey data in .csv format. Digital web tracking data in .csv format.
Description of data collection	Data were collected using a non-probability quota sampling method, so that the sample reflects the characteristics of the general population in each country in terms of region, gender and age. Sample recruitment and the data collection process were carried out by Netquest. Respondents were selected from a population of panellists from Netquest's metered panels who have – knowingly and consensually - digital tracking solutions installed in at least one of their devices, which allowed us to collect information about their online behaviours in addition to their survey responses. The overall participation rate among those who were invited ranged from 34.8% in Argentina to 77.9% in Chile, and the overall completion rate among those who participated ranged from 26.8% in Chile to 57.5% in Italy. Once participants accepted to participate and their responses collected, the data were cleaned to conform to ISO procedures. In particular, some interviews were discarded either because the socio-demographic profiles did not match the quotas established in the initial design (gender, age, region or locality size), because the time a respondent took to complete the whole survey was at least 20 percent lower than its estimated duration, or because individuals failed to pass an attention check or ‘trick’ question aimed at confirming that the participant was paying attention.
Data source location	Institution: Universitat Pompeu Fabra City/Town/Region: Barcelona, Catalonia Country: Spain
Data accessibility	Repository name: Open Science Foundation Data identification number: DOI 10.17605/OSF.IO/3T7JZ The data protocol & codebook, as well as the questionnaires for each of the three waves, are available at the TRI-POL project OSF website (https://osf.io/3t7jz/?view_only=22e669dfd9a946d5b706e0efcd584d7c).

## Value of the Data

• The survey datasets consist of a three-wave micro-panel survey implemented with a consistent methodology and questionnaire across five different countries: Spain, Portugal, Italy, Argentina, and Chile. Because of the cross-country panel design, the data allow us also to estimate individual-level variation (within variation) as well as across-individual differences (between variation), both over time and across countries.
• This project also includes datasets containing digital trace data collected using a combination of tracking technologies that the interviewees installed in their different devices. These data have allowed us to create a set of variables on individuals’ behaviours and exposure to information received via digital and social media.
• The survey data can be easily matched with the digital trace data. In the materials on the project website, there is a document entitled “FAQs – TRIPOL digital trace datasets” that has information on how to merge the datasets. The merging of these datasets results in a unique comparative dataset.
• To the best of our knowledge, the online survey data contain the most exhaustive list of indicators of affective and ideological polarisation to date, allowing for the construction of many individual-level indicators of polarisation.
• Those researching electoral behaviour can benefit from these data to understand the micro-foundations and contextual factors that could alter affective political polarisation in today's democracies, especially regarding the influence of digital media consumption, and social media activity.

## 1 Objective

The objective of this dataset is to provide researchers with high-quality data on political polarisation, political trust, party competition, and political communication. To our knowledge, the datasets contained in this project include the most exhaustive batteries of survey questions on polarisation, both ideological and affective, as well as trace data on individuals’ online behaviours, which more accurately reflects online activities than self-reported measures. Both the trace and the survey data in the TRI-POL project are available for five different countries over three different waves, providing researchers with much greater leverage in analysing over time and across contexts.

## 2 Data Description

Within the TRI-POL project there are two groups of datasets. The first group consists of survey data collected from the panel participants in each of the five countries, available in Excel (.xlsx) format. The survey data files are names “TRI_POL_XX” where the XX corresponds to the country code (AR for Argentina, CL for Chile, ES for Spain, IT for Italy, and PT for Portugal). In the second group are datasets for each of the five countries with the digital trace data collected from participants’ online activities (available in .csv format). Each of these datasets is named “Passive_meter_data_XX_WX” where the first XX corresponds to the country code and the second X corresponds to the wave number. There are fifteen digital tracking datasets in all: three datasets, one for each wave, for each of the five countries. Both groups of datasets as well as additional supplementary files such as questionnaires and data protocols are available (in .pdf format) at the project's Open Science Framework (OSF) site (DOI 10.17605/OSF.IO/3T7JZ).

The survey datasets contain data from micro-level online panel surveys of the voting age population in five countries, with three waves carried out over a six-month period between 2021 and 2022. The survey datasets include three embedded experiments (one per wave). Table 1 shows a list of the main variables in the survey data organised by topics. The surveys include questions on socio-demographic characteristics, self-reported voting behaviour and intentions, non-electoral political participation, membership and involvement in a broad array of social and political associations and organisations, an equally ample catalogue on media consumption and internet and social networks usage, a rich battery of measures of political and affective polarisation, a wealth of variables tapping into political opinions, attitudes, and orientations, a series of indicators on trust in political parties and institutions, as well as in people of several relevant social groups, and a profuse set of evaluations of the economic and political situation. Most of these questions are asked in all waves, enabling both the study of their evolution and the impact of their changes at the individual level on other variables. The battery of political and affective polarisation indicators is especially rich in that it includes sentiments towards candidates, voters, and relevant social groups, as is the battery of opinions on salient policy-issues and questions on the placement of respondents and the most important political parties on the left-right ideological dimension. In order to easily distinguish between the different groups of variables, there are prefixes attached to the variable names. The prefix ‘g’ indicates global variables, which apply to all waves, such as the panellists’ unique identification numbers. Sociodemographic variables are identified by an ‘s’ prefix and ‘esmP’ indicates experimental variables.

**Table 1:** List of main variables by type and topic with variable name and label.

Survey variables	Indicators of affective polarisation

	(a) Feelings towards supporters of different political parties (all waves) (p16 battery, Feelings towards VOTERS) (b) Feelings towards leaders of different parties (all waves) (p17 battery, Feelings towards LEADER) (c) Feelings towards different social, religious, or economic groups (waves 1 and 3) (p15 battery, Feelings towards GROUP) (d) Social distance (wave 3) (p42 battery, Child marriage in/out-party) (p43 battery, Hire in/out-party member) (p44 battery, In/out-party friendship) (e) Adjectives describing most-liked and least-liked voter group (open-ended) (waves 2 and 3) (p41 battery, Description of most-liked/least-liked voters) Indicators of ideological polarisation (a) Left-right scale (self-location) (all waves) (p12) (b) Location of parties in the left-right scale (all waves) (p13 battery) (c) Identification with the ideological labels “Left”, “Right” and “Center”, (all waves) (p40 battery). (d) Issue-based polarisation (all waves) (p15 battery) Emotions provoked by political leaders (all waves, all leaders) (a) Hopeful (p17 battery, LEADER hopeful) (b) Proud (p17 battery, LEADER proud) (c) Angry (p17 battery, LEADER angry) (d) Fearful (p17 battery, LEADER fearful) (e) Indifferent (p17 battery, LEADER indifferent) (f) Disgusted (p17 battery, LEADER disgusted) Partisan identification battery (all waves) (p33 battery)Probability of voting for different parties (PTVs) (all waves) (battery p36)Trust in institutions (all waves) (battery p19. The list changes from country to country) (p18Indicators of social and particularised trust (a) Trust in different social groups (waves 2 and 3) (p18 battery) (b) General trustworthiness of people (all waves) (p20 battery) Political interest (all waves) (p1, Political Interest)Characteristics of and opinions toward democracy (all waves) (a) Importance of different characteristics of democracy (p5 battery) (b) Preference for and satisfaction with democracy (p8, Preferred political regime) (p9, Satisfaction with democracy in COUNTRY) (c) Support for authoritarian-oriented actions (p7 battery) Political efficacy (waves 1 and 3) (p4 battery)Political participation (online and offline) (waves 1 and 3) (p34 battery) (a) Signed a petition (p34a, Signing a petition) (b) Boycotted products (p34b, Boycotting products) (c) Worn campaign badges or stickers (p34c, Displaying campaign propaganda) (d) Participated in demonstrations (p34d, Participating in demonstrations) (e) Participated in rallies (p34e, Participating in political rallies) (f) Contacted a politician (p34f, Contacting a politician) (g) Posted political opinions online (p35g, Posting political opinions on social media) Voting behaviour (a) Probability to vote in next elections (waves 1 and 3) (p35, Probability to vote in upcoming general elections) (b) Vote probability for different political parties (all waves) (p36 battery, Probability to vote PARTY) (c) Vote intention (all waves) (p37, Preferred party for upcoming election)
	Political knowledge (waves 1 and 3) (p38 battery)Main problems facing the country (waves 1 and 3) (p3_, Main problem in COUNTRY)Opinions on current situation on different issues (waves 1 and 3) (p10a, Unemployment) (p10b, Education) (p10c, Health) (p10d, Immigration), (p10e, Pensions), (p10f, Corruption) (p10g, Social inequality) (p10h, The COVID-19 pandemic) (p11, Satisfaction with current national government).Media consumption (self-reported) and trust in different media (waves 1 and 3) (p21 battery)
	(a) Print newspapers (p21a, Print newspapers political news source) (p21h, Print newspapers trust) (b) Online newspapers (p21b, Online newspapers political news source) (p21i, Online newspapers trust) (c) Radio (p21c, Radio political news source) (p21j, Radio trust) (d) Magazines (p21d, Magazines political news source) (p21k, Magazines trust) (e) Blogs (p21e, Blogs political news source) (p21l, Blogs trust) (f) Television (p21f, Television political news source) (p21m, Television trust) (g) Social media (p21g, Social media political news source) (p21n, Social media trust) Political discussion (waves 1 and 3) (p22 battery) (a) In person (p22 battery, Family) (p23 battery, Friends) (b) On social media (p25 battery) Main sources for obtaining information (waves 1 and 3) (a) Frequency (p 26 battery) (b) Trust (p27 battery) Discussing of politics on messaging services (waves 1 and 3) (a) Frequency and agreement (battery p28) Information sharing on messaging services (waves 1 and 3) (batteries p29 and p30)Opinions on fake news (all waves) (p31 battery) (a) Fake news on mainstream media (p31a, Fake news on mainstream media frequency) (b) Fake news on social media (p31b, Fake news on social media frequency) (c) Fake news on messaging apps (p31c, Fake news on messaging apps) (d) Fake news in face-to-face conversations (p31a, Fake news in face-to-face conversations frequency) Sociodemographic variables (all waves) (a) Gender (s1_, Gender) (b) Age (s2_, Age) (c) Employment status (s8_, Employment status) (d) Income and financial status (s9_, Feelings about household income) (s11a_, Concern about paying bills) (s11b_, Concern about reducing standard of living) (s11c_, Concern about employment) (s11d_, Concern about debt/mortgage) (s12_, Net household income) (s13_, Financial satisfaction) Sociodemographic variables (wave 1) (a) Locality (g11, HABITAT_) (b) Education (g10, EDUCATION_) (c) Marital/civil status (s5_1, Marital/civil status) (d) Number of children (s6_1, Number of children) (e) Religiosity (s14_1, Religiosity) (f) Household characteristics (s7_1, Number of cohabitants)

Experimental variables	Experiment on the influence of exposure to social media on political attitudes (wave 1). All the information is contained in the Panel survey DATAPROTOCOL (a) Followed Twitter accounts (esmp0a_1) (b) Agreement to participate (esmP0b_1) (c) Random selection of type of account to follow (esmP0c_1) (d) Final compliance (esmP1_1) (e) Respondent selected account (esmP2_1) (f) Whether accounts previously followed (esmP3_1) (g) Topics discussed (esmP4_1) (it varies among countries) (h) Agreement with opinions (esmP5_1) (i) Tone of opinions (esmP6_1) (j) Trust in account (esmP7_1)
	Polarisation, populism, and interpersonal trust game (wave 2) (a) Understanding of game 1 rules (esmP8_2, esmP9_2 and esmP10_2) (b) Willingness to participate (esmP0c_2) (c) Points given to other player (game 1) (esmP11_2) (d) Populist/non-populist treatment (esmP12_2) (e) Polarizing treatment (esmP13_2) (f) Unifying treatment (esmP14_2) (g) Populist dispositional framing (esmP15, esmP16_2) (h) Non-populist situational frame (esmP17_2 and esmP18_2) (i) Points given after preceding treatments players (esmP19_2) (j) Points given player in game 2 (reciprocity) (esmP20_2) Conjoint experiment on social sorting (wave 3) (all included in batteries esmP12XXXX_3)General Neighbour profile (although it might vary in some of the countries): (a) National and subnational identity trait (b) Language (c) Ideological trait (d) Country of birth (e) Type of family (traditional nuclear or non-traditional) (f) Partisan trait (g) Level of Education (h) Environmental behaviour (i) Pet ownership (j) Religion trait (k) Level of Politicisation

Passive meter variables	Online behaviour variables (Waves 1 and 3). All this information is contained in the passive meter data protocol documents (one for each country) under Passive Meter (3 waves). The names of the variables change from country to country depending on the URLs tracked. The main variables are the following: (a) Average number of visits and seconds spent on the Internet in general (b) Average number of visits and seconds spent on the top 50 most popular news media outlet websites (c) Average number of visits and seconds spent on political / national / international / regional and opinion news in the top 50 most popular news media outlet websites (d) Average number of visits and seconds spent on each of the top 50 most visited news media outlet websites e) Average number of visits and seconds spent on political / national / international / regional and opinion news in each of the top 50 most popular news media outlet websites (f) Average number of visits and seconds spent on social media networks in general (g) Average number of visits and seconds spent on each social media network individually: Facebook, Twitter, Instagram, TikTok, Snapchat, YouTube, WhatsApp, Messenger. (h) Average number of visits and seconds spent on the Twitter profile of a selected number of politicians, political parties and other political institutions Methodological variables (all waves) (a) Number of devices tracked and not tracked (b) Identification of whether a participant does not have all devices tracked (c) Identification of whether an individual has error-induced non-observations Type of devices, OS, and browsers used (all waves)

In each of the three survey waves, there is a different embedded experiment that provides additional information on the causality behind affective polarisation: one on 1) media effects, and social media exposure; 2) a trust game exploring the effect of priming political polarisation or populist political frames, and (3) a conjoint experiment to assess the effect of partisan and ideological polarisation on social and political tolerance.

The web tracking datasets include variables measuring the daily number of visits or seconds spent on a set of given webpages or groups of webpages, as well as to specific content (e.g., political articles) within those webpages. Specifically, it includes measures about the average number of visits or time spent on the top 50 most popular news media outlets in each country, as well as on the most used social media networks globally. It also includes the visits and seconds spent on URLs defined as opinion articles, news in general and national, regional, international, and political news. In addition, the datasets also include variables about the visits and seconds spent on specific Twitter profiles (the ones used for the experimental design). All variables included in the datasets are repeated three times: one variable documenting the average behaviour during the 15 days before starting the survey, one documenting the average during the 15 after starting the survey, and a general one documenting the average behaviour during the entire month of tracking. The prefixes “PRE”, “POST” and “ALL” are used to distinguish the tracking period used to compute the averages.

## 3 Experimental Design, Materials and Methods

### 3.1 The Survey Design

In the sections below, we explain in more detail the methodological strategy for both the survey and digital web tracking data collection. In Section 2.1, we begin by discussing the timing of the waves in each country, summarised in Table 2, followed by the recruitment, participation and retention of respondents across waves. Table 3 provides information on the number of respondents in each sample and what proportion of those respondents were tracked. Then we have an explanation of recruitment, acceptance, rejection, and overall participation rates, including why certain participants were rejected. These figures are presented in Table 4, followed by the attrition rates in Table 5. We then conclude Section 2.1 with a brief description of the experiments that are embedded in the different waves of the survey. In Section 2.2, we move into more specific information on the collection of the digital tracking data. One of the issues we address is how we handled the undercoverage of devices, the proportions of which are found in Table 6, which is due to one or more devices of a participant not being covered by the tracking application. We also explain the issue of error-induced non-observations in the variables of the digital tracking dataset, with the proportion of respondents with these types of non-observations in Table 7.

**Table 2:** Timing of the waves.

	Wave	Begin	End	Days	Gap
Spain	Wave 1	23/09/2021	18/11/2021	57	n.a.
	Wave 2	01/12/2021	09/01/2021	40	12
	Wave 3	31/03/2022	21/04/2022	22	81
	All	23/02/2021	21/04/2022	119	

Portugal	Wave 1	23/09/2021	12/11/2021	51	n.a.
	Wave 2	01/12/2021	06/01/2022	36	18
	Wave 3	31/03/2022	22/04/2022	23	84
	All	23/09/2021	22/04/2022	100	

Italy	Wave 1	23/09/2021	19/11/2021	58	n.a.
	Wave 2	01/12/2021	08/01/2022	39	11
	Wave 3	31/03/2022	20/04/2022	21	82
	All	23/09/2021	20/04/2022	118	

Argentina	Wave 1	23/09/2021	15/11/2021	53	n.a.
	Wave 2	01/12/2021	08/01/2022	39	15
	Wave 3	31/03/2022	22/04/2022	23	82
	All	23/09/2021	22/04/2022	115	

Chile	Wave 1	23/09/2021	18/11/2021	57	n.a.
	Wave 2	01/12/2021	08/01/2022	39	12
	Wave 3	31/03/2022	20/04/2022	23	82
	All	23/09/2021	20/04/2022	119	

*Source*: own elaboration.

*Notes*: Days = The number of days during which survey responses were collected. Gap = time elapsed, in days, from the last day of data collection of the previous wave to the first day of response collection of the current wave; n.a.: not applicable, since in the first wave there is no previous wave with respect to which a time gap may be calculated.

**Table 3:** Full sample and tracked sample in each country wave.

	Wave	Wave 1	Wave 2	Wave 3
Spain	Full Sample	1289	1162	1080
	Tracked Sample	993	930	842

Portugal	Full Sample	1028	905	818
	Tracked Sample	818	749	670

Italy	Full Sample	1231	1116	999
	Tracked Sample	842	786	688

Argentina	Full Sample	1316	1114	979
	Tracked Sample	1127	1000	848

Chile	Full Sample	1337	1084	921
	Tracked Sample	958	830	686

**Table 4:** Invitations, participation, and data cleaning in the three waves for Spain.

	Spain				Portugal				Italy				Argentina				Chile			
Wave	Wave 1	Wave 2	Wave 3	Sum	Wave 1	Wave 2	Wave 3	Sum	Wave 1	Wave 2	Wave 3	Sum	Wave 1	Wave 2	Wave 3	Sum	Wave 1	Wave 2	Wave 3	Sum
Rejected and accepted invitations																				

Invited	11136	1289	1162	13587	8002	1028	905	9935	5922	1231	1116	8269	32225	1316	1114	34655	13587	1337	1084	16008
Rejected	5042	90	53	5185	3238	86	53	3377	2271	91	89	2451	22360	121	98	22579	3238	176	123	3537
Accepted	6325	1199	1109	8633	4764	942	852	6558	3651	1140	1027	5818	9865	1195	1016	12076	10349	1161	961	12471
Participation rate	56.8%	93.0%	95.4%	63.5%	59.5%	91.6%	94.1%	66.0%	61.7%	92.6%	92.0%	70.4%	30.6%	90.8%	91.2%	34.8%	76.2%	86.8%	88.7%	77.9%

Discarded and completed interviews																				

Accepted	6325	1199	1109	8633	4764	942	852	6558	3651	1140	1027	5818	9865	1195	1016	12076	10349	1161	961	12471
Discarded	5036	37	29	5102	3736	37	29	3802	2420	24	28	2472	8549	81	37	8667	9012	77	40	9129
Declined	223	0	0	223	303	0	0	303	276	0	0	276	2053	0	0	2053	4432	0	0	4432
ISO unmet	32	4	8	44	17	4	8	29	19	5	12	36	48	2	2	52	52	1	3	56
Incomplete	2975	33	20	3028	1628	33	20	1681	1357	15	14	1386	3948	66	29	4043	2896	75	37	3008
Invalid	0	0	1	1	0	0	1	1	2	0	0	2	0	1	1	2	0	1	0	1
Closed	859	0	0	859	1239	0	0	1239	249	4	2	523	1346	12	5	1363	494	0	0	494
Quota full	947	0	0	947	549	0	0	549	517	0	0	517	1154	0	0	1154	1138	0	0	1138
Completed	1289	1162	1080	3531	1028	905	823	2756	1231	1116	999	3346	1316	1114	979	3409	1337	1084	921	3342
Completion rate	20.4%	96.9%	97.4%	40.9%	21.6%	96.1%	96.6%	42.0%	33.7%	97.9%	97.3%	57.5%	13.3%	93.2%	96.4%	28.2%	12.9%	93.4%	95.8%	26.8%

*Source*: own elaboration.

*Notes*: Accepted invitations constitute the starting point of the lower panel of the table, and are in turn disaggregated between interviews that are completed and those that are discarded on accounts of different criteria: a. *Declined participation*: a small fraction of those who had initially accepted the invitation (overall, less than 1,9%) declined to participate after learning the goals of the questionnaire or the institution responsible for the study. b. *ISO unmet*: some interviews (overall, 0,4% of those who had accepted to participate) where discarded because they failed to meet ISO quality standards. Participations are labelled as “ISO unmet” when they fail to meet at least one of the following criteria: 1) the information on gender or age provided in the survey is not consistent with the one previously available in the database; 2) the response time is considered as fraudulent, i.e., the survey is completed in less than 20% of the estimated time; 3) the individuals failed to pass an attention check or ‘trick’ question. c. *Uncompleted interview*: a somewhat larger number of interviews (overall, 836, i.e., 7,2% of those who had accepted to participate) were discarded because they were not fully completed. d. *Invalidated interview*: only 1 case in all waves of those who had accepted to participate were discarded due to software issues (i.e. the program did not save the answers to some questions) e. *Closed*: one of the largest groups of discarded interviews (859 or 7,4% of those who had accepted to participate) was made up of those who completed the interview but did so only after the field had been closed. f. *Quota full*: finally, 947 interviews (8,2% of those who had accepted to participate) were discarded because the quota for a respondent's profile had already been filled.

The completion rate (i.e., the proportion of those who successfully completed the survey after accepting the invitation) ranges from 13.0% in the first wave to 97.0% in the third and fourth one, with an average of 69%.

**Table 5:** Wave attrition.

	Wave	Wave 1	Wave 2	Wave 3
Spain	Completed	1289	1162	1080
	Consecutive completion	n.a.	1162	1080
	Immediate permanence rate	n.a.	90.1%	92.9%
	Cumulative completion	1289	1162	1080
	Cumulative permanence rate	100%	90.1%	83.8%

Portugal	Completed	1028	905	823
	Consecutive completion	n.a.	905	823
	Immediate permanence rate	n.a.	88.0%	90.9%
	Cumulative completion	1028	905	823
	Cumulative permanence rate	100%	88.%	80.1%

Italy	Completed	1231	1116	999
	Consecutive completion	n.a.	1116	999
	Immediate permanence rate	n.a.	90.7%	89.5%
	Cumulative completion	1231	1116	999
	Cumulative permanence rate	100%	90.7%	81.2%
Argentina	Completed	1316	1114	979
	Consecutive completion	n.a.	1120	979
	Immediate permanence rate	n.a.	85.1%	87.9%
	Cumulative completion	1316	1114	979
	Cumulative permanence rate	100%	85.1%	74.4%

Chile	Completed	1337	1084	921
	Consecutive completion	n.a.	1084	921
	Immediate permanence rate	n.a.	81.1%	85.0%
	Cumulative completion	1337	1084	921
	Cumulative permanence rate	100%	81.1%	68.9%

*Source*: own elaboration.

*Notes*: Completed = accepted – (declined + ISO unmet + incomplete + invalid + closed + quota full). Immediate permanence rate = consecutive completion / completed. Cumulative permanence rate = cumulative completion / completed in wave 1. n.a.: not applicable.

**Table 6:** Proportion of participants undercovered in terms of device, in all countries for waves 1 and 3.

Italy			Portugal		Spain		Argentina		Chile	
Device	W1	W3	W1	W3	W1	W3	W1	W3	W1	W3
	76.1	76.7	76.5	75.3	70.3	66.9	70.0	67.9	73.7	72.7
N	842	688	818	675	992	844	1,127	848	958	693

Unweighted proportions.

**Table 7:** Proportion of participants with error-induced non-observations, out of all the tracked participants.

	Italy		Portugal		Spain		Argentina		Chile	
	W1	W3	W1	W3	W1	W3	W1	W3	W1	W3
Facebook	10.5	30.5	10.6	39.5	11.1	22.8	9.8	37.5	10.9	30.2
Twitter	23.0	15.0	17.7	15.8	14.7	17.7	16.1	25.2	21.1	26.7
Avg. News outlets	9.0	13.6	18.8	25.4	11.8	15.3	10.0	16.3	17.5	24.1
N	842	688	818	675	992	844	1,127	848	958	693

#### 3.1.1 The Survey Panel Design and the Timing of the Waves

The survey data are comprised of a three-wave online panel survey of the voting age population conducted between September 2021 and April 2022 in Spain, Italy, Portugal, Chile, and Argentina. Table 2 displays the timing of the waves for each country.

#### 3.1.2 The Survey Administration and Data Collection

The survey was administered by Netquest using their large online non-probabilistic population representative panel. Netquest (https://www.netquest.com/es/home/encuestas-online-investigacion) is an online people-based data collection company with over two decades of experience. Founded in Barcelona, Netquest currently conducts public opinion studies in 27 countries in Europe and the Americas. To do so, the company relies on online opt-in panels of people who are willing to participate in surveys and to share data about their online activity. Netquest currently works with various market research companies, public institutions, and universities worldwide. Specifically, data was collected using Netquest's metered panels. The Netquest metered panels provide a pool of individuals with digital tracking solutions installed in at least one of their devices, which allows us to complement their survey answers with information about their online behaviours. When the panellists agree to join the metered panel, they must install the meter in at least one device (PC, tablet, or smartphone) and start sending information (passively) to Netquest to become part of the metered panel. Participants receive more incentives if they install the meter in more devices (up to three).

#### 3.1.3 The Recruitment and Data Cleaning Process

For the recruitment of participants, a non-probability quota sampling method was applied, ensuring that the sample reflects the characteristics of the general population of each country in terms of region of residency, gender, and age. The quotas were derived from official statistics of each country. Respondents were selected from a population of panellists which already had the behavioural tracker installed at least six months before the study. Panellists from the Netquest metered panels have – knowingly and consensually – installed digital tracking solutions in at least one of their devices. Due to the difficulty in filling some of the specific cross-quotas with participants from the metered panel, in some cases the tracked participants were supplemented with non-metered panellists. Table 3 shows the number of participants in each country and wave that had the meter installed in at least one mobile (smartphone or tablet) or PC device compared to the full sample of survey respondents.

In the Data Protocols of the five country studies found on the OSF project website is information on the main socio-demographic characteristics of respondents by panel wave. The socio-demographic features of the participants are remarkably stable across the surveys and very close to the official statistical records.

For each country wave, Table 4 shows the number of invited participants, those who accepted the invitations, and those who failed to complete the questionnaire due to various reasons. The overall acceptance rate to participate ranged from 34.8% in Argentina to 77.9% in Chile. Participation rates are lowest for the first wave in all countries. In accordance with Netquest's standard procedures, the original data retrieved from the participants were cleaned to conform to ISO procedures. In particular, some interviews were discarded either because the socio-demographic profiles did not match those in the database in terms of gender or age, because the time a respondent took to complete the whole survey was at least 20 percent lower than its estimated duration, or because individuals failed to pass an attention check or ‘trick’ question aimed at confirming that the participant was paying attention. The combined number of interviews dropped due to any of these ISO criteria being unmet was remarkably low, ranging from 0.4% in Chile, Argentina, and Portugal, 0.5% in Spain and 0.6% in Italy. A somewhat larger number of interviews was discarded because they were incomplete, i.e., they had been started, but not finished, or were invalidated because the program did not save the answers to some questions (ranging from 24% in Italy and Chile to 35% in Spain). Finally, surveys were also removed because they had been completed after the data collection window had closed; or because the quota for a respondent's profile had already been filled (only wave 1). After taking these different situations into account, the effective number of completed surveys across countries oscillated between 13% and 34% in wave 1 and 93% to 98% in waves 2 and 3.

#### 3.1.4 Wave Attrition

To illustrate the rates of attrition across waves, Table 5 displays the number of respondents who completed questionnaires in each wave (row 1) and each pair of consecutive waves (row 2), the immediate rate of permanence from one wave to the next (row 3), the number of respondents who completed a wave's questionnaire and all the former ones (row 4), and the corresponding cumulative rate of permanence (row 5). The second wave is nested in the first, in that respondents of the second wave are a subsample of the first wave. Hence, the second wave's figures of row 1, row 2 and row 4 of each country are identical (in the case of Spain n_2_ = 1,162). Likewise, the third wave is nested in the second wave and therefore also in the first (n_3_ = 1,080). The immediate rates of permanence in row 3 capture the proportion of panellists in each wave who completed the survey in the next one. These rates are considerably high, ranging from 81% to 93%. The cumulative rates of permanence in row 5 capture the percentage of first-wave panellists who completed each wave; hence, the higher they are, the lower the attrition in the panel, which is one of the main concerns with micro-panel survey data. The figure for the cumulative rate of permanence of the third wave indicates that the percent of those who completed the first, second, and third waves varies between 69% and 84%.

The three waves in each country were designed to be successively nested. For example, in Spain, the 1,289 completed interviews in wave 1 is also the cumulative number of completed interviews at this stage. Wave 2 was effectively nested in wave 1. Therefore, all those who completed wave 2 (1,162) had also completed wave 1. This means that 1,162 is also the figure of *consecutively completed interviews* (i.e., of those who completed the current wave, in this case, wave 2, and the immediately previous wave, in this case, wave 1). Moreover, 1,162 is also the number of *cumulatively completed interviews* (i.e., of those who completed the current wave and all the previous ones). Likewise, wave 3 was nested in wave 2, meaning that the number of completed interviews in wave 3 (1,080) is also the number of consecutively completed interviews at this stage and, given that wave 2 was in turn was nested in wave 1, it is also the number of cumulatively completed interviews. This nesting strategy was used in each of the five countries.

#### 3.1.5 Basic Strategy for DK/NA

In dealing with responses that indicate uncertainty (i.e. “don't know”, “no option” or “decline to answer”), we follow recent literature on minimizing item nonresponse without adding additional error [1], [2], [3] or violating ethical norms of voluntary participation. In line with recommendations from Derouvray and Couper [4], we provide respondents with the option of skipping a question, while reminding them of the importance of their responses and request that they confirm they would like to skip the question and continue with the survey. We did not provide respondents with a “don't know” option except for questions that require some specific knowledge, where we expected such a response could be accurate and appropriate, such as questions on placing parties on the left-right scale or questions on political knowledge. More details on the strategy for dealing with nonresponse can be found in a previous *Data in Brief* article by the PI, Mariano Torcal, and his research team [5].

#### 3.1.6 The Experiments Embedded in the Different Waves

A different survey experiment was embedded in each of the three waves of the survey in all five countries of the study, maintaining as much consistency in the content of the experiments as possible across cases. More information on all these experiments can be found at https://osf.io/3t7jz/?view_only=22e669dfd9a946d5b706e0efcd584d7c.

(a) *Wave 1:* An experiment to measure the influence of exposure to social media on political attitudes
 The experiment embedded in the first wave of the TRI-POL survey was intended to test the effect of exposure to different Twitter accounts on a set of relevant political attitudes, such as political interest, affective and ideological polarisation, and political trust. Participation was restricted via invitation. Specifically, respondents were invited to follow one or two Twitter accounts from a list provided to them over a period of seven days. Two experimental groups were created with different lists of Twitter accounts. Using a computer algorithm, participants were randomly assigned either to the first group with a list containing accounts of political leaders of the main parties, or the second with a list of institutional accounts. After seven days, respondents who participated in the experiment were re-contacted, answered some questions about their exposure to and the content of the selected Twitter accounts, and completed the survey questionnaire about their political attitudes and opinions. Information was collected with the behavioural tracker to verify respondents’ activity on Twitter.
(b) *Wave 2:* An experiment on “polarisation, populism, and interpersonal trust in five countries”
 This study examines the effects of priming political polarisation or populist political frames on political polarisation as measured in interpersonal trust discrimination via behavioural games (i.e. trust games) and measures of political affect (feeling thermometers). Via simple randomisation, respondents were assigned to one of 5 groups: Control, Polarising Treatment, Unifying Treatment, Dispositional Issue Frame (populist) and Situational Issue Frame (non-populist).
(c) *Wave 3:* A conjoint experiment on social sorting
 There is increasing attention in the comparative literature to the origins and consequences of affective polarisation, a phenomenon referring to citizens’ growing sympathy towards co-partisans and antagonism towards supporters of other parties. Partisan animosity is reflected in a reluctance to engage with opposing partisans in non-political settings, lower levels of general social trust, as well as discriminatory behavior. Affective polarisation tends to be fueled by a strengthening of political and social identities and, in particular, the increasing alignment of social identities along party lines, i.e. ‘social sorting’. However, most of the comparative research exploring how social and political identities reinforce affective polarisation is based on observational studies, which does not allow testing the causal impact of these identities. This study tackles this limitation by using conjoint experiments in five multiparty systems which ask respondents to choose among hypothetical profiles of families moving to live next door, whose attributes varied along a number of characteristics including partisan support, ideology, ethnicity, immigration status/country of birth, sexual orientation and other country-specific relevant features. We have also included some “placebo” attribute (factor) such as “pet owner/no pet owner” that could work as a baseline to estimate the different effects of certain factors in selecting a specific neighbor profile. Asking respondents to choose between neighbors aims to capture social intolerance - i.e. the unwillingness to accept persons or groups with values and behaviors different from one's own by means of a co-existence –, which constitutes a fundamental threat to social cohesion.

### 3.2 The Web Tracking Design

#### 3.2.1 General Approach

Data about the participants’ online behaviours were collected for the 15 days prior to and after the participants started each survey wave. The behaviour tracker captured each URL (or app for mobile devices) accessed by the panellists, with timestamps for when the panellists first visited the URL, and the number of seconds in which the URL remained active in the browser. A URL was considered active when it was the one being displayed in the browser, meaning that other URLs that may be open in other tabs were not considered to be active. The number of active seconds was measured as the time between the URL (or app) first becoming active in the browser (i.e., displayed to the respondent) and a different URL (or app) becoming active in the browser. A visit was defined as any opened URL lasting one second or more.

TRI-POL researchers did not have access to the raw data with information about all URLs and apps visited by panellists, and their respective timestamps, to minimise any potential ethical concern linked with this project. Instead, a list of variables and guidelines on how to compute them was developed and sent to Netquest to implement. The guidelines can be checked in a document on the OSF project website. Netquest created and delivered several anonymised structured datasets, which complied with our specifications. Those databases were then processed by members of the TRI-POL project to create the datasets here described i.e., three separate datasets for each country, one for each wave. The data collection, processing and analysis approach was designed following the best practices recommended by Bosch and Revilla [6] in the Total Error framework for digital traces collected with Meters (TEM).

#### 3.2.2 Tracking Technologies Used

Participants were tracked on iOS and Android mobile devices, and Windows and MAC computers, using the tracking solutions provided by Wakoopa (https://www.wakoopa.com/). Specifically, Windows and MAC devices were tracked with desktop apps and/or web browser plug-ins, android devices through apps and iOS devices through manually configured proxies. Information about which technologies were used to track participants was requested from Netquest, which is provided in the databases. Table 1 of the passive meter data protocols of each country on the OSF project website provides more information about the characteristics, benefits, and limitations of the different technologies used.

#### 3.2.3 Tracking Undercoverage

All the variables in the web tracking datasets measure behaviours at the individual level (e.g., how much time someone spends reading news articles). Nonetheless, to achieve a complete vision of what a participant does online, we would need to track all the devices that they use to go online. If this is not achieved, only a partial image of a participant's online behaviour is observed, which can lead to errors such as underestimation of univariate estimates. This is known as tracking undercoverage [7].

Given that we were using a sample of participants who were already being tracked by Netquest, it was beyond our control to make sure that every participant was being fully covered. Following Bosch and Revilla's [7] recommendations in the Total Error framework for digital traces collected with Meters (TEM), we report the proportion of participants affected by tracking undercoverage.

To identify which participants were not tracked on all the devices they use to go online, we follow the best practices outlined by Bosch and Revilla [8]. We needed two pieces of information: which devices were tracked, and which devices participants used to go online. The first piece of information was obtained using paradata. In terms of paradata, we were able to identify the technology with which participants were being tracked, the type of device, the OS, whether it was a tablet or smartphone. The second piece of information was obtained by asking participants questions about which devices and browsers they used to access the internet during the 15 days before the first survey wave. See questionnaires on the OSF project website.

Combining both sources of information, we were able to identify when an individual was not fully covered in terms of device. Table 6 shows the following proportion of individuals with at least one device not being tracked, for waves 1 and 3.

#### 3.2.4 Dealing with Non-Observations

The variables in the web tracking datasets represent the time or number of visits that we observed individuals doing some specifically defined behaviours. These variables were created by Netquest, querying information from the entire raw dataset of all the URLs and apps that participants visited during the tracking period. For instance, a query could search the number of times that a specific participant ID had entered the webpage “elpais.com”. During this data extraction process, only available tracked traces could be used. For those cases in which no traces were observed (e.g., visits to elpais.com), the only reportable information was that no observation was found containing the queried information. Given the non-reactive nature of metered data, nonetheless, clear and transparent guidelines are needed when deciding what value to attribute to non-observations. Specifically, a lack of behaviour can be due to a true absence of behaviour (i.e., the participant never visited the defined URLs / apps in the query) or a failure to capture the data (e.g., because behaviours happened in non-tracked devices).

When tracking errors are present, especially tracking undercoverage, it is not possible to clearly discern when a lack of observed behaviours should be treated as real (e.g., 0 seconds visiting Facebook) or as a missing (i.e., a real behaviour happened, but we did not observe it) without auxiliary information. To account for this, and knowing that our dataset was affected by errors, we implemented an approach to identify when a specific observed lack of behaviour in the TRI-POL database was true or induced by errors, following the TEM's recommendations [7]. The approach followed this step-by-step process:

1. For participants with all devices and browsers tracked, which we identified thanks to the information presented in Section 3.2.3, we considered their non-observations as real. We coded those as 0 (i.e., 0 visits / 0 seconds).
2. For those participants identified as partially untracked, we considered their non-observations as dubious.
3. Dubious non-observations were then identified as true or not, using auxiliary information. Specifically, we asked participants whether they had visited, during the 15 days before the survey, some URLs/apps of interest with another device apart from the devices that we knew they were being tracked with. The question was personalised for each participant depending on the devices tracked according to the paradata. If they said “yes” for any domain, we considered their lack of behaviour as error (coded as -2, labelled as “Error-induced lack of behaviour”). If they said “no” we considered it as a true lack of behaviour (hence, as 0 seconds or visits). Asking this for all the variables in the dataset would have made the survey too long. Thus, we decided to ask this question only for the most important variables for most papers, the ones related to the consumption of Facebook, Twitter and the 10 most popular news media outlets in each country.
4. For all the other variables in the dataset, when a participant was identified as partially untracked, we considered their non-observations as uncertain (coded as -1, labelled as “Uncertain lack of behaviour”), given our impossibility of discerning whether the lack of behaviour was true or induced by errors.

For the specific variables with a clear distinction between real non-observations and error-induced ones (Facebook, Twitter and the ten most-visited news outlet of each country), we propose the following:

• True lack of observed behaviours: leave them as 0, which is their current value.
• Error-induced non-observations: re-code them as missing and exclude those participants from your analyses. Nonresponse weighting approaches can be used to re-adjust the sample.

The information used to identify non-observations as error-induced comes from self-reported data, which can also be affected by errors. Therefore, some participants might as well be wrongly misclassified as missing when following our approach. Users can decide to consider those non-observations as real, being mindful that the decision will most likely inflate the measurement errors of their results. For all the other web tracking variables, which only distinguish between real and uncertain non-observations, researchers need to decide whether they treat these non-observations as real (i.e., 0 seconds/visits) or induced by errors (i.e., missing). Although we cannot provide advice on what to do with these non-observations, all the research published so far has treated them as real non-observations (i.e., 0 seconds/visits). Regardless of the treatment, it is recommended to properly inform readers and reviewers about the proportion of participants which might have dubious non-observations treated as real. To have a better idea of the potential prevalence of this issue, Table 7 shows the proportion of participants with error-induced non-observations for the variables measuring the consumption of Facebook, Twitter and the top 10 news outlets in each country.

## Ethics Statements

The TRIPOL study and all of its data collection and storage protocols were approved by the Institutional Committee for Ethnic Review of Projects of the Universitat Pompeu Fabra (CIREP-UPF, application number 0181). All procedures performed in studies involving human participants were in accordance with the ethical standards of the institutional and national research committees and with the 1964 Helsinki declaration and its later amendments or comparable ethical standards. This article does not contain any studies with animals performed by any of the authors.

Informed consent was obtained from all individual participants included in the study.

## CRediT Author Statement

**Mariano Torcal:** Conceptualization, Funding acquisition, Investigation, Methodology, Supervision, Project Administration, Writing – original draft, Writing – review & editing; **Emily Carty:** Investigation, Validation, Writing – original draft, Writing – review & editing; **Oriol Bosch:** Data curation, Investigation, Methodology, Validation, Software, Writing – original draft; **Josep Comellas:** Conceptualization, Investigation, Validation, Writing – review & editing; **Zoe Thomson:** Data curation, Investigation, Methodology, Validation, Writing – review & editing; **Danilo Serani:** Data curation, Investigation, Methodology, Validation, Writing – review & editing.

## Declaration of Competing Interest

The authors declare that they have no known competing financial interests or personal relationships that could have appeared to influence the work reported in this paper.

## Data Availability

TRI-POL (Original data) (Open Science Foundation).
